# Management of a ciprofloxacin as a contaminant of emerging concern in water using microalgaebioremediation: mechanism, modeling, and kinetic studies

**DOI:** 10.1186/s12934-024-02591-y

**Published:** 2024-12-17

**Authors:** Heba Salah, Nabila Shehata, Noha Khedr, Khaled N. M. Elsayed

**Affiliations:** 1 Environmental Science and Industrial Development Department, Faculty of Postgraduate Studies for Advanced Sciences, Beni-Suef University, Beni-Suef, 62511 Egypt; 2 Botany and Microbiology Department, Faculty of Science, Beni-Suef University, Beni-Suef, 62511 Egypt; 3 Renewable Energy Science and Engineering Department, Faculty of Postgraduate Studies for Advanced Sciences, Beni-Suef University, Beni-Suef, 62511 Egypt

**Keywords:** Adsorption, Antibiotics, *C. vulgaris*, Ciprofloxacin, Contaminants of emerging concern (CEC), *Synechocystis* sp. PCC6803

## Abstract

Pharmaceutical residues, now recognized as a new category of environmental pollutants, have potentially risks to both ecosystems and human health effects. Recently, biosorption has emerged as one of the most promising strategies for managing these pharmaceutical wastes in water. Nevertheless, the environmental impact of the adsorbents presents a challenge to the advancement of this process. Therefore, the present study proposed two biosorbent: *Chlorella vulgaris* and *Synechocystis* sp. microalgae to manage Ciprofloxacin (CIP) in water. The experimental findings revealed that the optimal conditions for adsorption conditions are CIP initial concentration 4.0 mg/L and pH 5 and 3 for *Synechocystis*sp. and *C. vulgaris*, respectively. The adsorption process followed the Pseudo-second-order kinetic model. The main mechanism of biosorption is the complexation of CIP with carboxyl, hydroxyl, carbonyl, and amido groups which was confirmed by Fourier-transform infrared spectroscopy (FTIR), scanning electron microscopy (SEM) and energy-dispersive X-ray spectrometry (EDX) analyses. These analyses confirmed the presence of CIP on the surface of tested microalgal cells. These results indicated that the adsorption mechanism of CIP by *Synechocystis* sp. PCC6803 and *C. vulgaris* offers theoretical insights into the biosorption mechanisms of pharmaceutical residues.

## Introduction

Conventional water treatment systems have been shown to provide inadequate treatment of contaminants of emerging concern (CEC) [1]. The increasing worldwide contamination of freshwater with a manifold of pharmaceutical residues threatens aquatic organisms and human health. The environmental effects of pharmaceuticals, antibiotics, and disinfectants are of increasing concern [2]. The CEC has posed raising concerns recently. They are increasingly discharged in water and wastewater at worryingly high levels and being treated ineffectively in drinking water and wastewater treatment systems. The CEC can be classified as pharmaceuticals, personal care products, pesticides and industrial chemicals [3]. Due to the inevitable environmental release, antibiotics have been detected in global water which brings challenges to not only targeted bacteria but also to the health of non-target species such as fishes, plants, and algae [4]. Wastewaters from animal husbandry, aquaculture, and the pharmaceutical industry are the major sources of antibiotics in the environment [5]. Pharmaceutical residues are responsible for a number of harmful pollutants, such as antibiotics [6].

Antibiotics are often found in various environments and can be extremely dangerous for both human health and ecosystems [7]. Pollutants not subject to regulation are increasingly found in wastewater discharges, due to modern consumption patterns. These compounds are generally referred to be CEC due to the potential effects of their existence in the water systems world wide. Pharmaceuticals, personal care products, industrial additives, insecticides, and a variety of chemical compounds have all been detected in wastewater [3, 8]. Antibiotics, including ciprofloxacin (CIP), are used to mitigate or cure microbial infections and illnesses in veterinary, human, and aquatic systems by targeting specific bacteria. These antibiotics continually enter the aquatic environment by multiple pathways, such as hospital wastewater and pharmaceutical wastewater, veterinary, human excretions, and sewers, reaching treatment facilities in amounts ranging from ng/L to μg/L [9]. The occurrence of CIP in the surface water could achieved 5.02 mg/L [10]. The emergence of antibiotic-resistant genes (ARGs) and antibiotic-resistant bacteria (ARBs), which cause 700,000 annual fatalities, arethe main issues connected to antibiotic-polluted water [11]. Due to their resistance to the specific antibiotics suggested for their therapy, ARBs are extremely difficult to be treated [12].

Ciprofloxacin is a significant pharmaceutical drug belonging to the fluoroquinolone (FQ) class that targets both Gram-positive and Gram-negative bacteria to treat serious illnesses. Its global emissions are primarily found in surface water, which accounts for 25% of the total emission, and municipal wastewater, which accounts for 58% of the total emission [13]. This family of antibiotics is extremely mobile in the aquatic environment due to its hydrophilic characteristics. Fluoroquinolone antibiotic ciprofloxacin is found in a variety of sources, including drinking water and WWTP effluents, due to its significant usage in both human and veterinary medicine [14]. Like other antibiotics, CIP can stack up in the cells of organisms and pose a major risk to human health. The successful removal of CIP is therefore given adequate consideration to their high levels in wastewaters, stability, resistance to decomposition, and possible ecotoxicity [15]. Antibiotic removal has been accomplished by different methods, including coagulation, membrane separation, advanced oxidation, adsorption, photocatalysis, electrolysis, and biological degradation. These methods have several drawbacks, including high energy and material costs and a secondary contamination from the addition of other chemicals. Adsorption, on the other hand, is the most adaptable and extensively utilized of these removal processes because of its great removal capacity, high efficiency, straightforward design, and simplicity of usage. In this regard, biosorption which relies on the ability of various types of live and inactive dead biomasses (heat, dried, chemically treated) to bind and concentrate contaminants from water-based solutions has emerged as an environmentally friendly, practical, and financially viable method for the removal of antibiotics [16]. An ecologically benign method with great promise for antibiotic elimination is microalgae-based wastewater treatment. The precise antibiotics and microalgae species used, however, determine how well CIP is removed by microalgae [7, 17].

Microalgae are photosynthetic eukaryotic or prokaryotic organisms that can grow single, in chains, incolonies or in filamentous forms. They can be found in a variety of ecosystems, including airborne, aquatic, and terrestrial habitats [18, 19] and easily adjust to varying environments [20]. Microalgae serve a significant role in the oxygen production in aquatic ecosystems, as well as an important element of the food chain [21]. They have attracted interest in the bioremediation research for their capacity to accumulate and eliminate antibiotics from contaminated water, and yielding important biomass [11]. The antibiotic removal effectiveness by adsorption process is strongly reliant on the adsorbent, which is often costly. Oxidation and photocatalysis are usually effective, but they require expensive chemical agents or catalysts, as well as they could generate secondary pollutants. In contrast, microalgae wastewater treatment is an effective biological process to remove antibiotics requiring minimal chemical agents [22].The biosorption efficiency depends essentially on the sorbent properties and pollutants structures [23]. Algal cell walls containa variety of polymer assemblages and functional groups that can facilitate the biosorption of pollutants on theirsurface [16].

Factors affecting antibiotic removal performance by microalgae are algal species, antibiotic classes and concentration, and algal growth conditions [22].

This study aimed to determine the biosorption capability of *Synechocystis* sp. and *C. vulgaris* for CIP at different concentrations. The selected microalgae species are used without modification in powder form at a constant concentration in the removal of CIP. A thorough investigation is conducted on process optimization by the adjustment of process parameters, such as time, pH, and starting concentration, in addition to the isotherm of adsorption and kinetic investigations.

## Materials and methods

### Ciprofloxacin

Ciprofloxacin, C_17_H_18_FN_3_O_3_, is supplied from Organo for pharmaceutical and chemical industries (ORGANO PHARMA), Egypt. CIP concentrations. Table 1 represents the chemical structure, molecular weight [16], and *p*Ka values of CIP [24].CIP is used in different concentrations with constant algal concentration. To measure the removal efficiency of CIP by microalgae, we prepare 0.1 g from selected microalgae species in 200 mL distant water (control sample).

**Table 1:** The main characteristics of CIP

*p*Ka values of CIP	5.9 ± 0.15 (carboxylic acid group) and 8.89 ± 0.11 (basic N-moiety)
Molecular weight	331.3 g/moL
Structure	

### Algae and Algal conditions

In BG11 cultivation medium, *C. vulgaris* was grown with the addition of NaCl 1%, while *Synechocystis* sp., which is sensitive to NaCl, was also grown in BG11 cultivation medium which consists of NaNO_3_ 1.5 CaCl_2_·2H_2_O 0.036 Ferric ammonium citrate 0.012 EDTA·Na_2_·2H_2_O 0.001 K_2_HPO_4_ 0.04 MgSO_4_·7H_2_O 0.075 Na_2_CO3 0.02 Trace metal solution 1 ml/l a H_3_BO_3_, 2.86 g/l; MnCl_2_·4H_2_O, 1.81 g/l; ZnSO_4_·7H_2_O, 0.222 g/l; Na_2_MoO_4_·2H_2_O, 0.39 g/l; CuSO_2_·5H_2_O, 0.079 g/l; Co (NO_3_)_2_·6H_2_O, 0.049 g/l [25], but without the addition of NaCl. Fluorescent light was used for a 12 h light/12 h dark cycle at 20 ºC.

### Biosorption studies

The monitoring was carried out by taking 5 mL aliquots of medium for the determination of residual CIP concentration. All samples were centrifuged before analysis in the Universal Centrifuge Model: PLC-036 GEMMYCO (Taiwan). The maximum absorbance for CIP was inspected by scanning between 200 and 400 nm using a UV–Vis spectrophotometer 1800 UV-2600 (Shimadzu, Japan), and the maximum absorbance was found at 400 nm. All experiments were carried out in triplicate and the average results were reported. All graphs were carried out with the originprogram. For the equilibrium time, the adsorption of CIP was carried out at the optimum adsorption pH with 0.25 g of the adsorbent in 500 mL of CIP solution (10 and 20 mg/L), and stirring at 300 rpm. The adsorption capacity was evaluated for 26 h at room temperature.

To evaluate the effect of CIP initial concentration on the adsorption of CIP onto both microalgae, triplicate adsorptions were carried out for microalgae mass of 0.01 g and 20 mL of solution at concentrations ranged from 5–25 mg/L. After the adsorption, the samples’ adsorption capacity was evaluated.

### Adsorption isotherm modeling

The adsorption isotherm can be used to determine the biosorbent’s capability as well as the adsorption behavior required to remove the pollutant [26]. Langmuir, Freundlich, Dubinin-Radushkevich, Baudu, and Fritz-Schlunder biosorption isotherms were used in the current study to investigate the kinetic performanceof each of *C. vulgaris*’s and *synechocystis*’s in the removal of CIP. Adsorption models in a nonlinear form are statistically more dependable compared to models in a linear form [27].To determine the equilibrium adsorption capacity, Eq. 1 is used [28]:

1 $${q}_{e}=\frac{v\left({C}_{i}-{C}_{e}\right)}{m}$$

where q_e_ is theequilibrium adsorption capacity (mg/g), V refers to the sample's volume (L), C_i_ represents the initialconcentration of the solute (mg/L), C_e_ is the equilibrium concentration of the solute (mg/L) and m is the quantity of adsorbent used (g). Equation 2 is used to determine the percentage of contaminant removal [29].

2 $$\text{Removal \% }=\frac{{C}_{i}-{C}_{e}}{{C}_{i}}\times 100\%$$

## Results and discussion

### Effect of pH

**Fig. 1:**
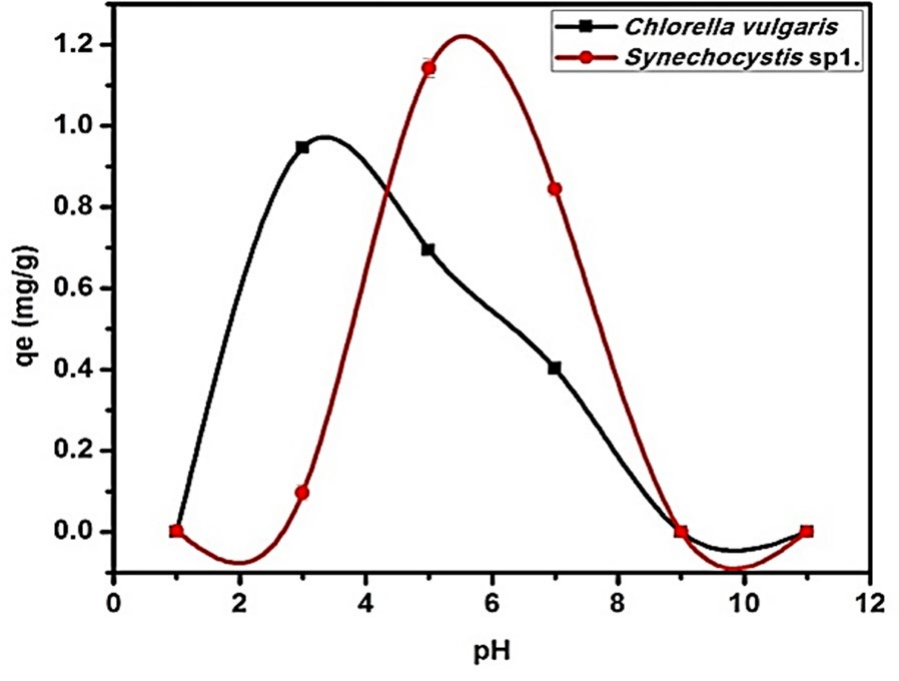
Effects of pH solution on the CIP biosorption in *C. vulgaris* and *Synechocystis* sp. at an initial concentration 20 mg/L and room temperature 20 °C

To investigate the effect of pH on the adsorption of CIP, 20 mg/L ofCIP solution was mixed with 0.5 g/L microalgae by using a shaker for 12 h at different pH values ranging from 3.0 to 11.0. The optimum pH values for the biosorption CIP onto *C. vulgaris*and*Synechocystis*sp.is 3.5 and 5.5, respectively which corresponds to removal efficiency of 90%. The optimal pH is vital because it affects ionization degree, adsorbent surface charge, and speciation of the adsorbate [30, 31]. Two *p*Ka values of CIP Table 1: for the basic-N moiety is 8.89 ± 0.11 and for the carboxylic acid group is 5.90 ± 0.15 [24].The acid dissociation constant (*p*Ka) of CIP is less than 6.0 when it is in its cation form because the amine group has been protonated, and it is more than 8.7 when it is in its anion form because the carboxylic group has lost a proton. The majority of CIP molecules are zwitterionic species, and their pH range is 6.0–8.7 [32, 33]. *C. vulgaris* and *Synechocystis*sp. have a pH_ZPC_ of 3.0. Hence, when the pH increased from pH 1 to pH 3 Fig. 1, the removal of CIP increased because of improved electrostatic attraction which results from the opposite charge between the CIP and the microalgae. In contrast, at high pH, CIP removal was significantly reduced. This may be due to the zwitterionic nature of CIP. At higher pH pH > 5.9, both CIP and the algal biomass possess negative charges and the repulsion forces will be the dominant.

Figure 1 illustrates that pH ranges from 3 to 7 resulting in higher CIP adsorption because of hydrophobic interactions between functional groups on the waste surface of *C. vulgaris* and CIP are responsible for the mechanism of biosorption. Comparing the removal of CIP efficiency at different pH, the adsorption of CIP decreases due to increased pH. *p*Ka value of CIP was 8.7 for the amine group and the value of *p*Ka of CIP was 6.1 for the carboxylic acid group on piperazine moiety [34]. Due to the carboxyl group’s proton being removed, CIP is present in an anion form [35, 36]. CIP is a cation that is present in solutions with a pH lower than 6.1, but likewise, CIP is present in solution as a zwitterionic form when the pH of the solution ranges from 6.1 to 8.7. The removal of CIP increased when the pH was less than 6, for the reason that electrostatic charge on the algae surface and CIP [37]. However, CIP removal was significantly reduced at high pH. It may occur due to algae surface charging and the zwitterion nature of CIP. High removal efficiencies are the result of ionic interactions between the surface of the adsorbent and CIP in acidic solutions [32, 37, 38]. The opposite charge between the electrostatic charging on the microalgae surface and the CIP causes electrostatic attraction, which leads to high removal efficiency.

### Effect of initial concentration

**Fig. 2:**
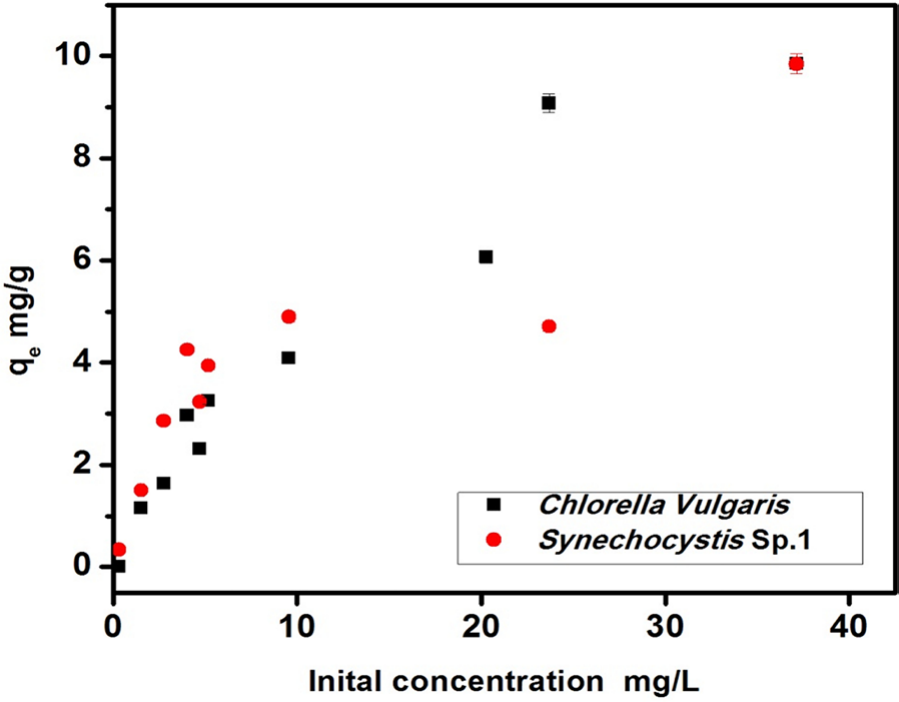
Effects of initial concentration on the CIP biosorption in *C. vulgaris* and *Synechocystis* sp. at room temperature 20 °C

According to Fig. 2, it can be observed that when the CIP initial concentration increased from 5 mg L^−1^ to 25 mg L^−1^, the adsorption capacity increased from 1.14 mg/g to 9.07 mg/g for *C. vulgaris* and from 0.33 mg/g to 9.84 mg/gfor *Synechocystis* sp. This is attributed to that the increase in CIP concentration in the solution, resulting in an increment in the difference between CIP concentration in the solution and CIP concentration at the microalga surface resulting in a greater driving force for mass transfer which promote the adsorption process at higher concentrations.

### Adsorption isotherm modeling

**Fig. 3:**
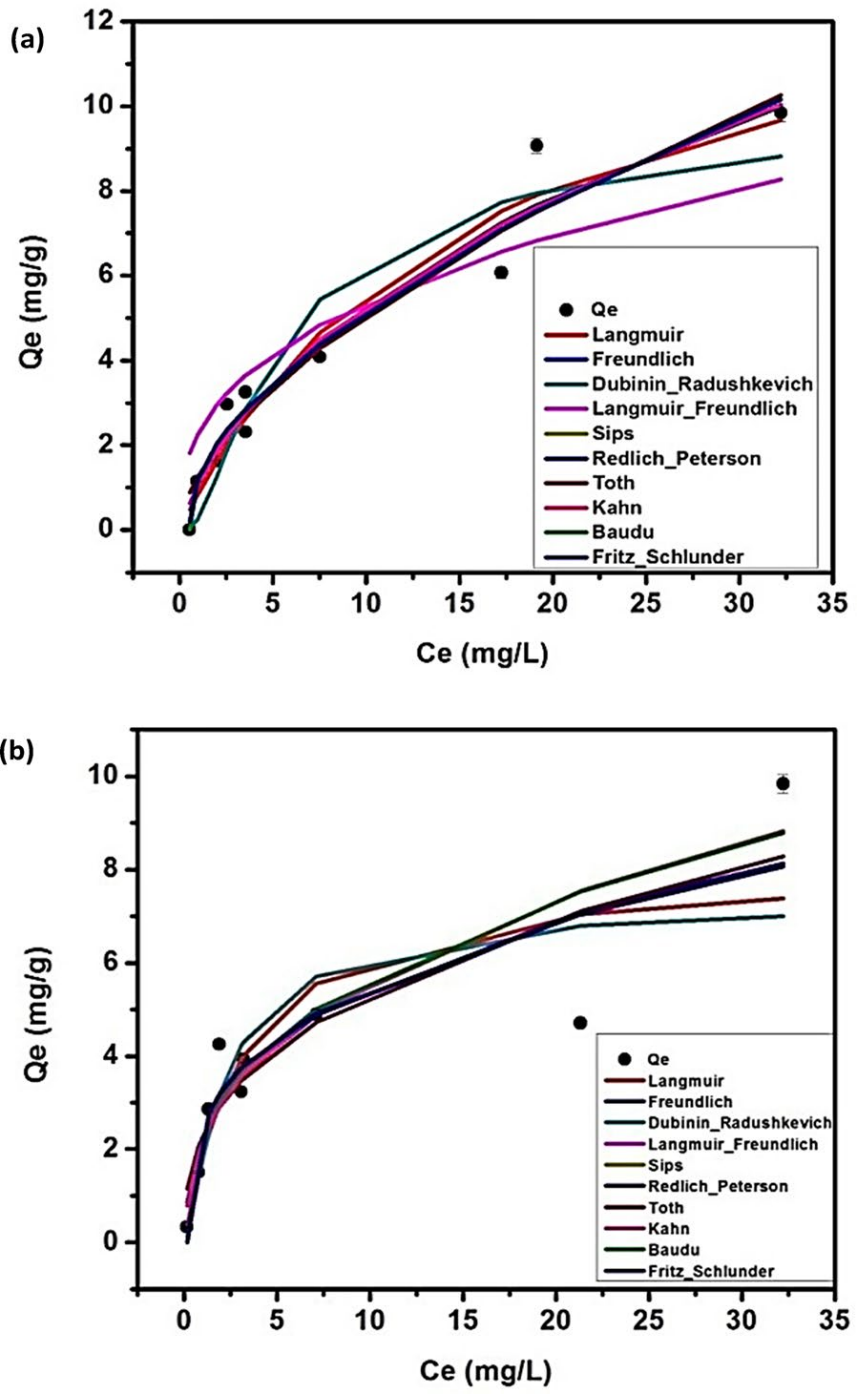
Adsorption isotherm modeling of CIP biosorption onto biomass (**a**) *Chlorella vulgaris* and (**b**) *Synechocystis* sp

Ten models have been investigated for the biosorption of CIP onto *C. vulgaris* and *Synechocystis*sp.1 Fig. 3. and Table 2. The results showed that Freundlich model isthe best to describe the CIP@*C. vulgaris* system where the calculated adsorption capacity is close to the calculated one in addition to a high correlation coefficient (R^2^ = 0.944), followed by Dubinin-Radushkevich and Langmuir with q_max_10.32 and 14.37 mg/g and R^2^ = 0.903 and 0.943, respectively [39]. The other models are not suitable for describing the CIP@*C. vulgaris* system such as Baudu, Redlich-Peterson, and Khan even with their high correlation coefficients (R^2^ = 0.953, 0.948, and 0.948, respectively) where the predicted q_max_ according to these models are less than the experimental one. Also, Sips and Toth models didn’t fit the data well where the calculated values of q_max_ according to these models are higher than the experimental one even with their high R^2^ values (0.944, and 0.944). Fritz-schlunder, and Langmuir–Freundlich can’t be used for the modeling of CIP@*C. vulgaris* system where the values ofR^2^are low (0.82 and 0.832, respectively) and the calculated values of q_max_ are far away than the experimental one.

**Table 2:** The parameters of the adsorption isotherm modelling for CIP@*C. Vulgaris* and CIP@*Synechocystis*sp. systems

Adsorption models		Parameter	*C. Vulgaris*	*Syn*. Sp	Adsorption models		Parameter	*C. Vulgaris*	*Syn*. Sp
2- parameters isotherm	Langmuir	q_max_(mg/g)	14.373	8.135	3-parameters isotherm	Redlich-Peterson	K_R_	1.567	12.0
		K_L_	0.063	0.303			a_R_	0.452	4.3
		R^2^	0.943	0.748			Β	0.630	0.686
	Freundlich	K_f_	1.283	2.286			R^2^	0.948	0.805
		1/n_F_	0.598	0.370		Sips	q_m_(mg/g)	11939.624	16276
		R^2^	0.944	0.800			Ks	0.000	0.000
	Dubinin-Radushkevich	q_max_ (mg/g)	10.322	7.452			1/n	0.598	0.370
		K_ad_	0.001	0.000			R^2^	0.944	0.800
		R^2^	0.903	0.726		Langmuir–Freundlich	*q*_MLF_(mg/g)	938.18	938.180
4-parameters isotherm	Baudu	q_m_(mg/g)	1.379	2.43			*K*_LF_	9.563	9.6E-08
		b_0_	76.783	998.2			*M*_LF_	0.372	0.372
		x	0.574	0.370			R^2^	0.832	0.800
		y	47.678	22.71		Toth	K_e_	23362	46048
		R^2^	0.953	0.86			K_L_	45336	32002
5-parameters isotherm	Fritz-schlunder	q_mFSS_(mg/g)	67.409	84.8			n	0.401	0.629
		K_1_	0.202	0.254			R^2^	0.944	0.800
		K_2_	9.924	8.305		Kahn	Q_m_(mg/g)	2.705	1.169
		m_1_	6.385	6.25			b_K_	0.486	6.695
		m_2_	5.808	5.93			a_K_	0.512	0.62
		R^2^	0.953	0.82			R^2^	0.948	0.86

For the CIP@*Synechocystis* sp. system, Freundlich is the best model to describe the system with acalculated q_max_ close to the experimental one and acceptable R^2^ (0.80).Followed by Redlich-Peterson (R^2^ = 0.805). Baudu, Sips, Langmuir–Freundlich, Toth, Fritz-schlunder, and Kahn failed to describe the CIP system where the predicted q_max_ values according to these models are far away from the experimental one. Although Langmuir and Dubinin-Radushkevich yield calculated q_max_ close to the experimental, however, the values ofR^2^arelow (0.748 and 0.726, respectively).

### Effect of time

**Fig. 4:**
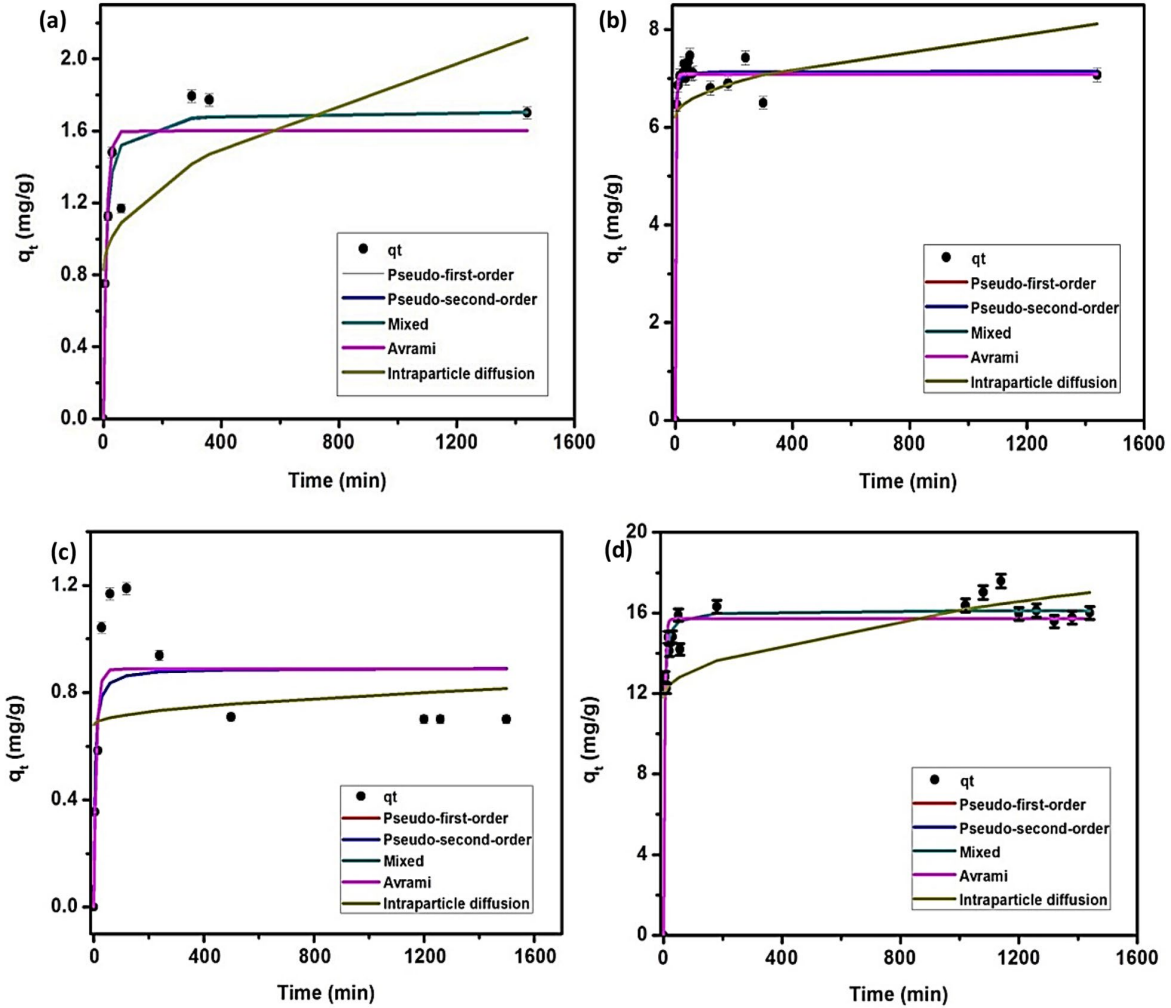
The kinetic modelling of the CIP biosorption onto *C. vulgaris* at (**a**)10 mg/L, (**b**)20 mg/L*,* and onto *Synechocystis* sp. at (**c**)10 mg/L, and (**d**)20 mg/L

The equilibrium time for the adsorption of CIP at initial concentration 10 mg/L onto *C. vulgaris* Fig. 4a shows that the adsorption capacity increases rapidly during the 30 min until it reaches q_t_1.48 mg/g, then there is a gradual increasein q_t_ (1.79 mg/g) up to 300 min, beyond this time there in no significant increase was noticed. Increasing the CIP initial concentration to 20 mg/L Fig. 4b, decrease the equilibrium time where the equilibrium occur after 60 min and the maximum q_t_was 7.11 mg/g. This is attributed to that increasing the CIP initial concentration increase the driving force for the adsorption to reach the equilibrium faster than that at lower concentration. For *Synechocystis* sp., and at CIP initial concentration 10 mg/L, Fig. 4c shows that increasing the contact time from 0 to 60 min increase q_t_ from 0 to 1.17 mg/g and increasing the time to 1440 min resulting in reduction in q_t_ (0.7 mg/g). This may be attributed to occurrence of partial desorption at longer time. At higher CIP initial concentration (20 mg/L), Fig. 4d shows that increasing the contact time from 0 to 180 min increase q_t_ from 0 to 16.31 mg/g and beyond this time, no significant change was recorded.

### Kinetics

The kinetic of the adsorption process yields significant insights to design a batch adsorption system and it also provides optimum operating conditions for full-scale operation. Therefore, the experimental results of CIP adsorption onto both microalgae were studied using Pseudo 1^st^order (PFO), Pseudo 2^nd^order (PSO), Avrami, Mixed 1st and 2nd (MFSO) and intraparticle diffusion models, whose results are represented in Fig. 4 and Table 3.

**Table 3:** The parameters of kinetic models describing the adsorption of CIP onto *C. vulgaris* and *Synechocystis*sp

Model	Parameters	*C.vulgaris*		*Synechocystis*sp.	
		Conc.10 mg/L	Conc.20 mg/L	Conc.10 mg/L	Conc.20 mg/L
Pseudo-first-order	q_e_ [mg/g]	1.600	7.083	0.887	15.715
	k_1_[L/mg]	0.094	0.478	0.100	0.253
	R^2^	0.890	0.981	0.708	0.923
Pseudo-second-order	q_e_[mg/g]	1.712	7.145	0.892	16.138
	k_2_	0.077	0.349	0.278	0.032
	R^2^	0.937	0.979	0.631	0.962
Mixed 1, 2-order	q_e_[mg/g]	1.710	7.098	0.887	16.124
	K	0.000	0.175	0.100	0.001
	*f*_2_	0.998	0.859	0	0.998
	R^2^	0.937	0.982	0.708	0.962
Avrami	q_e_[mg/g]	1.600	7.083	0.887	15.715
	k_av_	0.380	0.854	0.390	0.621
	*n*_av_	0.249	0.559	0.256	0.407
	R^2^	0.890	0.981	0.708	0.923
Intraparticle diffusion	k_ip_	0.033	0.050	0.003	0.137
	*c*_ip_	0.826	6.200	0.678	11.793
	R^2^	0.484	0.065	0.020	0.292

Four models; PFO, PSO, Avrami, and MFSO can describe the CIP@*C. Vulgaris* system, especially at the higher concentration of CIP following the order: MFSO (R^2^ = 0.982) > PFO (R^2^ = 0.981) and Avrami (R^2^ = 0.981) > PSO (R^2^ = 0.979) while in the lower concentration of CIP, MFSO (R^2^ = 0.937) and PSO (R^2^ = 0.937) are better than PFO (R^2^ = 0.890) and Avrami (R^2^ = 0.890).

On the other hand, the intraparticle diffusion model is not suitable for this system where the predicted data do not agree with the experimental one as well as R^2^ values are low (0.484–0.065). Forthe CIP@*Synechocystis*Sp.,PFO (R^2^ = 0.923), PSO (R^2^ = 0.962), Avrami (R^2^ = 0.923), and MFSO (R^2^ = 0.962) can fit the data at CIP initial concentration 20 mg/Lwell with excellent matching between the experimental and the predicted data in addition to high values of R^2^ while at lower concentrations(10 mg/L), the correlation coefficients decreased to 0.708, 0.631, 0.708 and 0.708 for PFO, PSO, MFSO and Avrami models, respectively. On the other hand, the intraparticle diffusion model is not suitable for CIP@*Synechocystis*sp. at both initial concentrations of CIP where the predicted values don’t agree with the experimental one in addition to low values of R^2^ Table 3.

### Comparative study

Table 4 list different microalgae species used to manage CIP in water.

**Table 4:** Ciprofloxacin removal by different microalgae species

Microalgae	Antibiotic concentration, removal rate and retention time	Removal mechanisms	Wastewatercategory	References
*Chlamydomonas mexicana*	2 mg/L and 13%, 11 d	Biodegradation, accumulation, and adsorption	Bold’s Basal medium	[40]
*Nannochloris* sp.	57 ng/L and 100%, 7 d	Direct photolysis	Water from Las Vegas wash	[41]
*Chlamydomonaspitschmannii*	2 mg/L and 1.6%, 11 d	Biodegradation, accumulation, and adsorption	Bold’s Basal medium	[40]
*Ourococcus multisporus*	2 mg/L and 2%, 11 d	Biodegradation, accumulation, and adsorption	Bold’s Basal medium	[40]
*Chlorella Vulgaris*	2 mg/L and 0%, 11 d	Biodegradation, accumulation, and adsorption	Bold’s Basal medium	[40]
*Chlamydomonas Mexicana*	2 mg/L and 56%, 11 d	Biodegradation, accumulation, and adsorption	Bold’s Basal medium + sodium acetate (4 g/L)	[40]
The mixture of algae-bacteria consortia in pilot high-ratealgae pond (HRAP)	1.31 mg/L and 20.1%, 24 h (8 h sunlight/16 h dark)	Photodegradation during daytime, and adsorption during nighttime	Real domestic wastewater	[42]

### FTIR

**Fig. 5:**
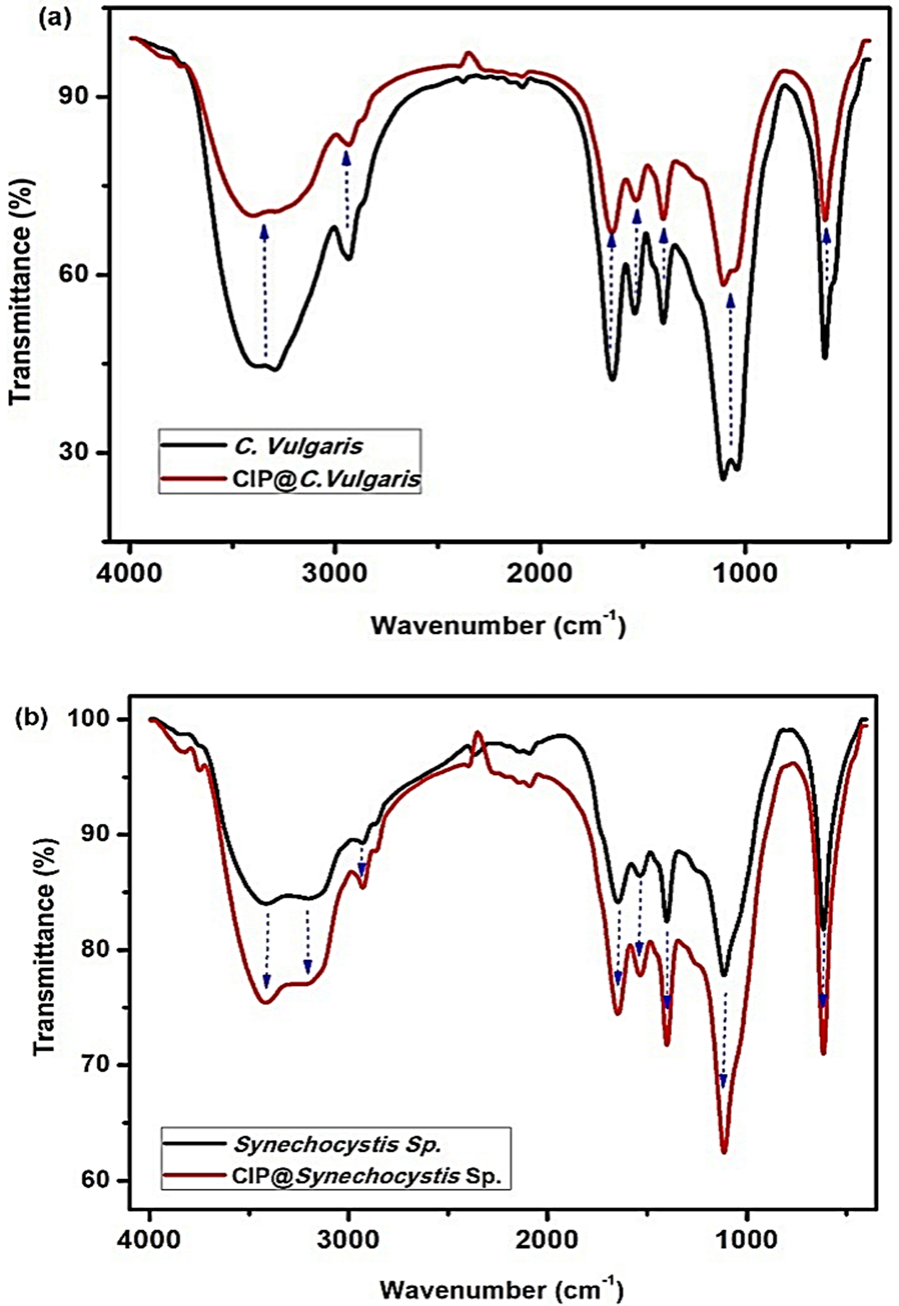
The FTIR spectra of (**a**) C. *vulgaris* biomass and (**b**) Synechocystis sp. biomass before and after CIP adsorption

Fourier transform infrared spectroscopy (FT-IR) is a common instrumental tool used for the identification of several functional groups of any organic material (liquids, solids, and gases) by the measurement and determination of its emission spectra or infrared absorption [43].The impact of CIP adsorption onto *C. vulgaris* and *Synechocystis*sp. on the change in theirchemical structures was detected via FTIR analyses Fig. 5. The results showed that stretching vibration of water molecules owning to the intermolecular bonding of OH- appear at 3293.452 cm^−1^ and 3414.190 cm^−1^for *C. vulgaris* and *Synechocystis*sp., respectively [44]. The asymmetrical (–C–H) and stretching (–C–H) vibration have been detected at 2933.767 cm^−1^and 1400.41 cm^−1^for *C. vulgaris* and 2929.20 cm^−1^for *Synechocystis *sp [45]. The band at2933.767 cm^−1^isbelonging to CH and CH_2_ groups of the aliphatic of carbohydrates lipids and proteins. The band at1646.01 cm^−1^is attributed to C = O group of amide Ib and of protein. The band of carbohydrate CO group is appearedat1041.408 cm^−1^. The amide II band is confirmed at1535.336 and 1539.176 cm^−1^ [46]. The presence of band at1539.176 cm^−1^ indicates the stretching vibration of N–H of amide II and the bending vibration of C–N.For *C. vulgaris*, the characteristic bands appear at 3293.452 cm^−1^ (stretching, N–H of protein) [45, 47], 1648.624 cm^−1^ (stretching, C = O of protein and C = C) [48], 1539.176 cm^−1^(bending, amide (N–H and C–H) and (C–N) stretching vibration of protein [49] and stretching, C = C) [49]. The band at1400.407 cm^−1^referred to stretching of C = C [49] while that at1108.482 cm^−1^may be attributed to the carbohydrate V (–O–C) of polysaccharides, nucleic acid, stretching of phosphodiesters carbohydrate [47] and alkyl stretching [45]. The band at 1041.408 cm^−1^is attributed to carbohydrate V(C–O–C) of polysaccharides and alkyl stretching. Moreover, the latter is confirmed by another band at 613.338 cm^−1^. For *Synechocystis *sp., the characteristic bands were detected at 1646.01 cm^−1^ (C = O highly conjugated [50] and the stretching vibration of amide I in proteins [48], 1535.336 cm^−1^(carboxyl group in salt from –COO–,the stretching vibration of amide II in proteins [48], 1400.593 cm^−1^(CH_3_) [50], asymmetrical C–H bending mode of –CO–CH_2_– or CO–CH_3_ groups [51], stretching vibration of C = O in the carboxyl group [52, 53], and1114.017 cm^−1^(C–O stretch and O–H bend in phenoxy structures, ethers [50].

After the CIP adsorption, there is no significant change in the two spectra of the microalgae except the variation in the intensity of the bands. This may refer to that the adsorption process occurred due to the presence of amide, hydroxyl, carboxyl, and carbonyl groups. The intensity of the bands after CIP adsorption decreased in the case of *C. vulgaris* Fig. 5a and increased in the case of *Synechocystis *sp. Fig. 5b. This may be attributed to the involvement of various functional groups on *C. vulgaris *in the attachment of CIP and the formation of new bands with higher density in the case of *Synechocystis *sp. which agreed with the SEM results and adsorption isotherm modeling.

### SEM

**Fig. 6:**
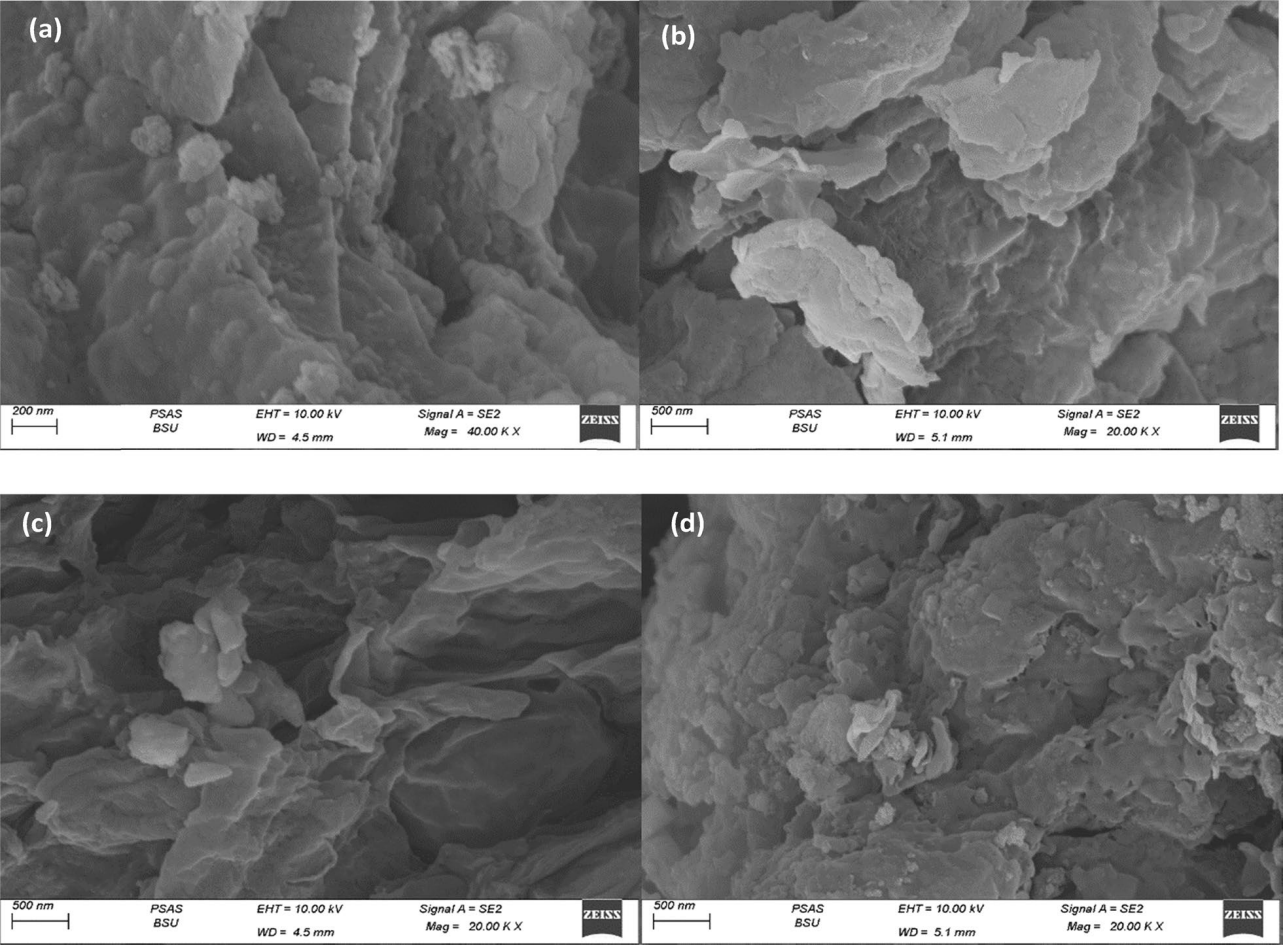
The SEM images of *C. vulgaris* (**a**) before and (**b**) after CIP biosorption and *Synechocystis* sp. (**c**) before and (**d**) after CIP biosorption

The surface morphology of the two biomasses before and after CIP adsorption was observed using SEM Fig. 6. The two biomasses exhibit heterogeneous surfaces and possess small cavities/crakes on their surfaces. Figure 6a confirms that *C. Vulgaris *is irregularly shaped, with a close, compact, and smoother structure, and after the biosorption of CIP Fig. 6b, it exfoliated which may be attributed to the attachment of CIP to specific functional groups onto the biomass in monolayer form which is agreed with the results of the adsorption isotherm modeling.

Figure 6c shows that *Synechocystis* sp. biomass has an irregular shape. After adsorption Fig. 6d, the surfaces of the cells were compact with some roughness. Also, it can be seen the aggregation of some attachments onto the surface owing to the precipitation or accumulation of CIP on the cavity on the cell surface which agreed with the modeling results suggesting that Freundlich isotherm is the predominant in CIP.

### Mapping and EDX

**Fig. 7:**
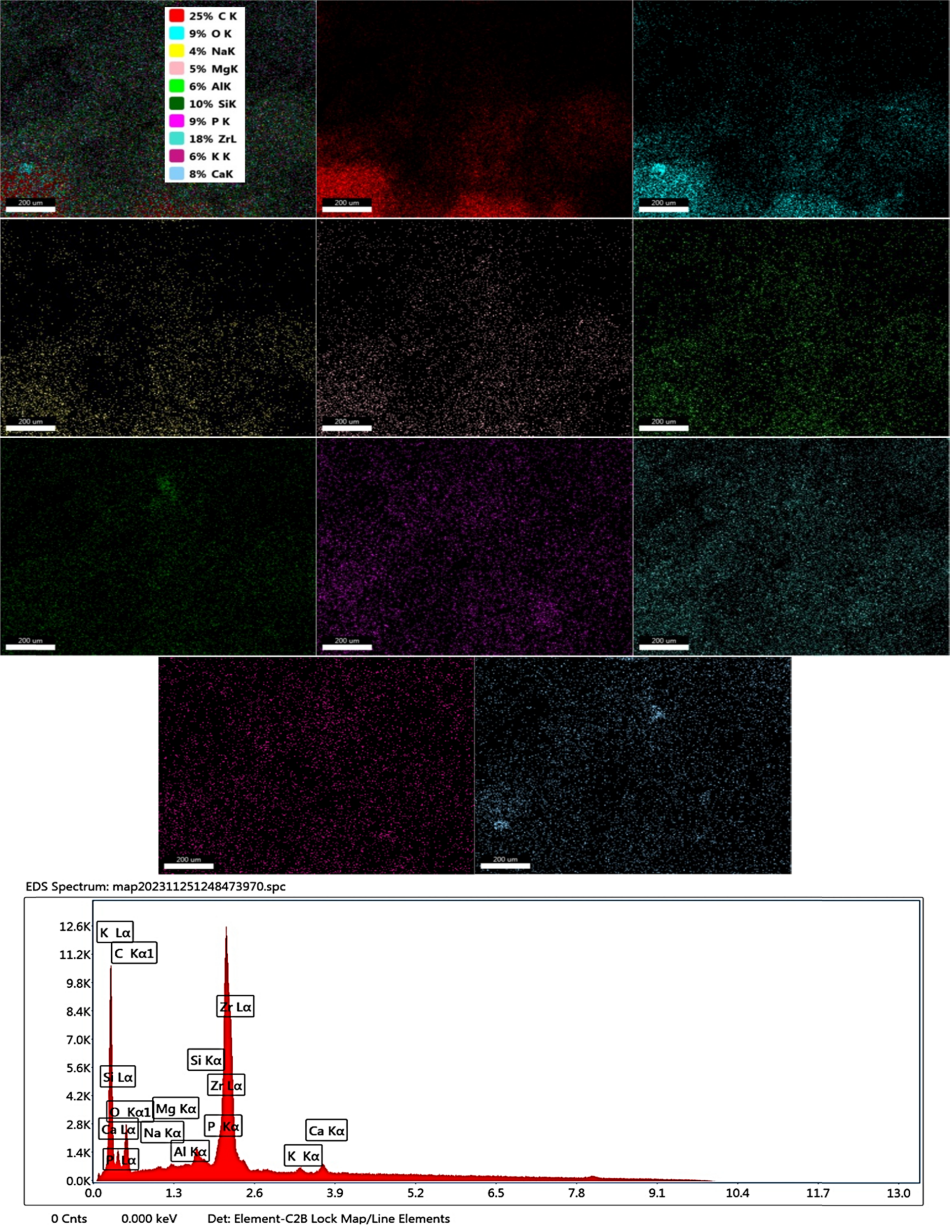
Mapping and EDX of *C. vulgaris* biomass

**Fig. 8:**
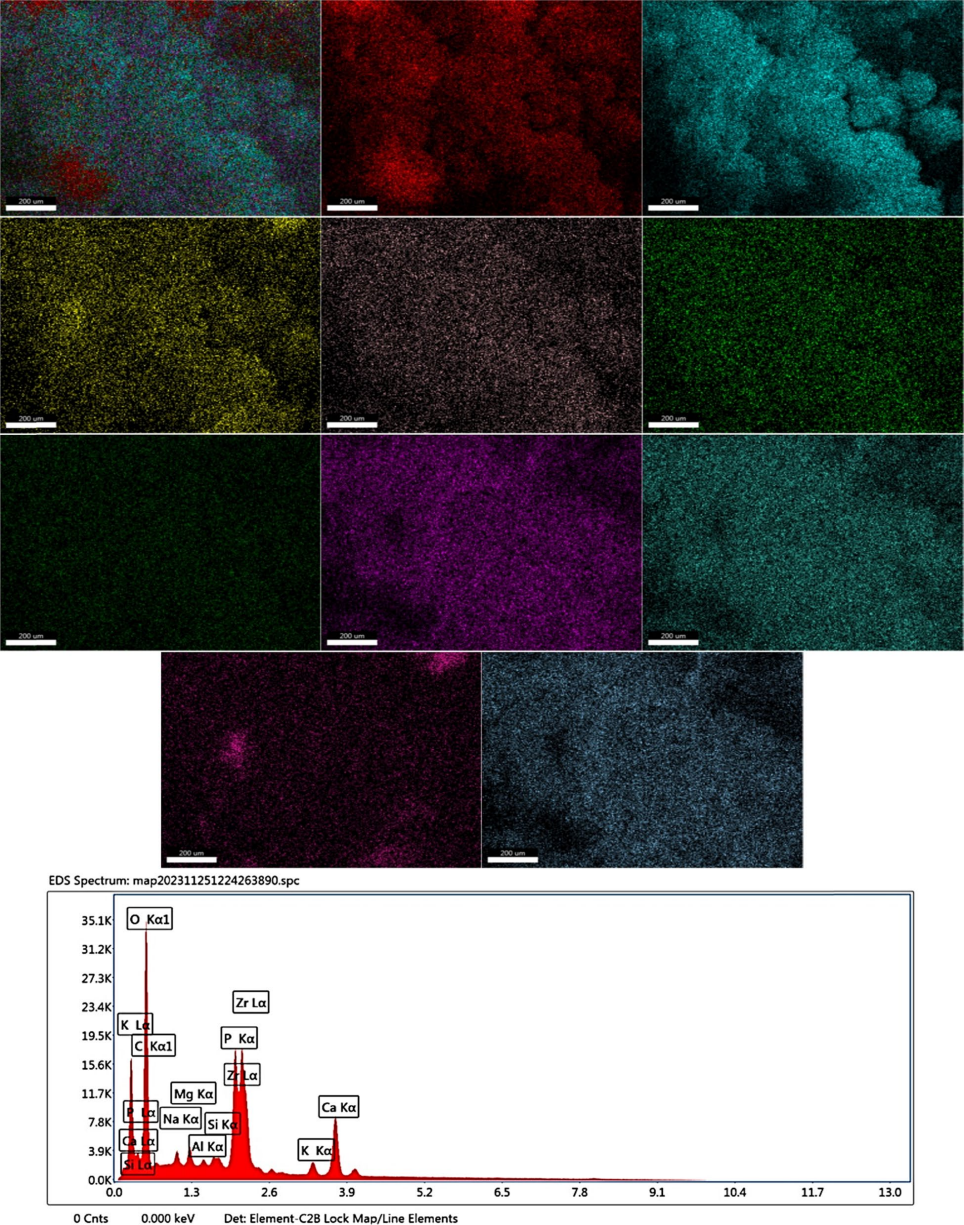
Mapping and EDX of *Synechocystis* sp.biomass

The elemental composition of the selected areas in SEM images of *C. vulgaris* Fig. 7 and *Synechocystis* sp. Fig. 8 was determined by Energy Dispersive X-Ray (EDX) Analysis. The EDX analytical data indicated that oxygen, carbon, nitrogen, and iron were present in the *C. vulgaris* Fig. 7 and *Synechocystis *sp. Fig. 8 microalgae. This result confirmed the successful incorporation of CIP molecules into the *C. vulgaris* and *Synechocystis *sp. algae [54].The study showed that applying microalgae successfully removes CIP compounds from contaminated water.

## Conclusion

In the current study, the removal of CIP was investigated from contaminated water by using microalgae as an adsorbent. The two types of algae used in the current research are *chlorella vulgaris* and *Synechocystis* sp. The effects of several factors on removing CIP by microalgae were tested (e.g., pH, CIP dosage, adsorbent concentrations, contact time, and temperature**).**The adsorption of CIP increases with an increase in CIP initial concentration and contact time, up to a definite limit. Based on isotherm data, the adsorption of CIP by microalgae follows Langmuir isotherm model. The kinetic data show that CIP adsorption fits second-order kinetic models depending on R^2^ values and the comparison of calculated and experimental q_e_ values.

## Data Availability

No datasets were generated or analysed during the current study.

## Abbreviations

CIP: Ciprofloxacin
*C. *vulgaris: *Chlorella vulgaris*
*Synechocystis* sp.1: *Synechocystis sp*. PCC6803
FQ: Fluoroquinolones
ARB: Antibiotic-resistant bacteria
ARG: Antibiotic-resistant gene
CEC: Contaminants of emerging concern
FLU: Flutamide
SEM: Scanning electron microscope
FTIR: Fourier-transform infrared spectrum
PZC: Point of zero charge
XPS: X-ray photoelectron spectroscopic
*q*_max_: Maximum adsorption capacity (mg/g)
PFO: Pseudo-first-order
PSO: Pseudo-second-order
MFSO: Mixed first andsecond order
*q*_e_: The amount of adsorbate in the adsorbent at equilibrium (mg/g)
*C*_O_: The initial equilibrium dye concentration (mg/L)
*C*_e_: The equilibrium dye concentration (mg/L)
V: The volume of solution, L
W: The mass of adsorbent used, g
K_L_: Langmuir isotherm constant (L/mg)
*K*_f_: Freundlich adsorption capacity (mg/g)
*K*_LF_: Langmuir–Freundlich equilibrium constant for heterogeneous solids
1/*n*_F_: Freundlich adsorption intensity
n: The empirical constant
k_1_: Pseudo-first-order rate constant, min^−^^1^
k_2_: The rate constant of pseudo-second-order model, g/(mg min)
